# Fibroblast activation protein inhibitor PET/CT as an emerging diagnostic modality in interstitial lung disease and other fibrotic conditions

**DOI:** 10.2478/rir-2024-0021

**Published:** 2024-10-21

**Authors:** Mihai Tudor Albu, Alexandru-Emil Matei, Jörg H. W. Distler, Frederik L. Giesel, Yuriko Mori

**Affiliations:** Department of Rheumatology, University Hospital Düsseldorf, Medical Faculty of Heinrich-Heine University; Düsseldorf, Germany; Hiller Research Center, University Hospital Düsseldorf, Medical Faculty of Heinrich-Heine University; Düsseldorf, Germany; Department of Nuclear Medicine, University Hospital Düsseldorf, Medical Faculty of Heinrich-Heine University; Düsseldorf, Germany; Institute for Radiation Sciences, Osaka University, Osaka, Japan

**Keywords:** fibrosis, fibroblasts, systemic sclerosis, interstitial lung diseases

## Abstract

Interstitial lung diseases (ILD) encompass a wide range of disorders characterized by alveolar inflammation and fibrotic tissue remodeling, marked by significant morbidity and mortality. Systemic sclerosis (SSc), among other connective tissue diseases, is a frequent cause of ILD. Assessment of pulmonary fibrosis is frequently constrained by the delayed manifestations of profibrotic activation of fibroblasts, which results in late macroscopic alterations detectable by standard imaging techniques such as computed tomography (CT) and magnetic resonance imaging (MRI) scans. 68Ga-labeled fibroblast activation protein inhibitors (68Ga-FAPI [fibroblast activation protein inhibitor]) are novel radionuclides used in the selective positron emission tomography/computed tomography (PET/CT) detection of profibrotic fibroblasts, a key player in fibrotic tissue remodeling. Application of 68Ga-FAPI in different target organs undergoing fibrosis, such as lung and heart, highlights its efficacy in detecting ongoing fibrotic processes, since FAPI tracer uptake has been correlated with clinical disease progression markers in SSc-ILD. This feature could enable physicians to detect subclinical fibrotic activity and tailor an individualised therapy plan on a case by case basis. The use of 68Ga-FAPI in ILD and other fibrotic conditions may emerge as a novel tool in future clinical practice for both activity monitoring and treatment optimisation. Other tracers tested in ILD of different etiologies have shown promising results and may in future also be considered for potential application in SSc-ILD.

## Introduction

Interstitial lung disease (ILD) is a heterogeneous group of pulmonary diseases characterized by inflammatory changes in the alveoli and fibrotic remodeling of the interstitium, accompanied by marked functional impairment.^[1]^ Rheumatic diseases such as systemic sclerosis (SSc) are a well-known cause of ILD, which in turn is a leading cause of morbidity and mortality in this patient group.^[2,3]^ Accurately assessing current disease activity is fundamental for therapy modifications, and effective imaging modalities have until recently been lacking in identifying progressive fibrosis.

Morphological tissue evaluation via radiographic imaging is currently the most widely used method in clinical routine: Serial computed tomography (CT) scans can detect macroscopic organ changes in comparison to baseline scans. This approach, however, fails to assess the functional process driving the structural change and the ongoing, underlying fibrotic activity. Accordingly, functional changes, *e.g*. in the course of therapy, are only visible after longer observation periods, which subjects the patients to the risk of overtreatment. The need for better disease insight has driven clinicians towards the employment of dual functional and structural imaging techniques in SSc.^[4,5]^

## Molecular Imaging in Interstitial Lung Disease

^18^F-Fluorodeoxyglucose (FDG) is the most commonly used tracer in clinical PET imaging. Before the emergence of fibroblast activation protein (FAP) tracer as a specific fibroblasttargeting agent, ^18^F-FDG-PET/CT had been investigated as a means to evaluate idiopathic pulmonary fibrosis (IPF).

Measured parameters such as metabolic lung volume and total lesion glycolysis were correlated with more severe disease and a worse prognosis,^[6]^ suggesting the efficacy of PET-based metabolic assessment of pulmonary fibrosis. One of the studies on IPF cohorts using FDG-PET showed significant correlation of tracer uptake with increasing pulmonary fibrosis, functional status and survival rate after lung transplantation.^[7,8]^ Another clinical study illustrated the ability of FDG-PET to detect inflammatory areas before the occurrence of morphological change.^[9]^ In the case of SSc-ILD, pulmonary uptake of ^18^F-FDG was positively correlated with fibrosis extent and inversely correlated with the diffusing capacity for the lung of carbon monoxide (DLCO) and the forced vital capacity (FVC).^[5]^ Moreover, there was a significant difference between those patients with progressive ILD compared to those without.^[5]^ However, ^18^F-FDG-PET shows higher tracer accumulation in tissues with increased glucose utilization including inflammation, which results in too unspecific imaging properties to be a valid diagnostic work-up of ILD.

## FAPI-PET Imaging

Fibroblast activation protein (FAP) is a membrane-bound type 2 serine protease belonging to the dipeptidyl peptidase 4 family, which is overexpressed on activated fibroblasts, but not on resting fibroblasts or other cell types. Recently, fibroblast activation protein inhibitors (FAPIs) have been developed that bind to FAP with high affinity. This specific binding allows the identification of foci involved in the ongoing fibrotic tissue remodeling, which is distinguished from healthy, unaffected tissue. Coupling of quinolone-based FAPI with DOTA chelators led to the introduction of FAPI-PET-tracers that enable visualization and quantification of activated fibroblasts in situ, with inherent diagnostic potential. Through fusion of FAPI-PET data with spatial CT data, increased fibroblast activity in affected tissues can be detected even in earlier stages and quantified accordingly.

The majority of the current publications on FAPI-PET focus on the oncological application in multiple clinical settings. Fibroblasts within the tumoral stroma undergo a transition towards an activated phenotype,^[10]^ which is characterized by the overexpression of FAP. Over 90% of epithelial carcinomas are reported to contain stromal fibroblasts exhibiting high FAP expression, providing a reliable target for molecular FAP imaging.^[10]^ Multiple studies have demonstrated the efficacy of FAPI-PET in depicting the extent of cancerous lesions more precisely, leading to more accurate oncological staging.^[10]^ In contrast to FDG, FAPI is independent of glucose metabolism, therefore reducing unspecific signal from highly glucose-consuming organs such as the brain, liver, and gastrointestinal tract.^[11]^ Although FAPI is not superior in all cancer entities.^[11,12,13]^ high sensitivity in multiple cancer detection, coupled with its low unspecific binding, makes FAPI-based PET/CT scans a promising tool in future oncological practice. It is also potentially available for theranostic applications.^[11]^

## FAP Targeting in Fibrotic Diseases

The role of fibroblasts in ILD progression has been well documented and many molecular pathways involved have been reported thus far.^[14,15]^ As previously described, FAP targeting enables physicians to assess loci of ongoing fibrotic remodeling, a hallmark of ILD pathogenesis. Multiple preclinical studies have demonstrated positive FAP tracer uptake in fibrotic lung tissue, such as in bleomycin-exposed murine model.^[16,17,18]^ Rosenkrans *et al*. have recently demonstrated that the Ashcroft scores of these mouse models, which is the scale used for quantifying the degree of fibrosis in Masson Trichrome’s stained lung tissue samples, correlated significantly with FAPI uptake, providing the basis of quantification using FAPI-PET.^[16]^

Based on these findings, several clinical evaluations of FAPI-PET imaging in ILD patients have been performed. In SSc-ILD, Bergmann *et al*. have shown that FAP tracer has accumulated in fibrotic areas of the lung compared to healthy controls and that higher FAPI uptake at baseline was associated with ILD progression, independent of the extent of involvement on high-resolution computed tomography (HRCT) scan and baseline FVC.^[19]^ In patients treated with the antifibrotic agent nintedanib, consecutive 68Ga-FAPI-04-PET/ CT scans correlated FAPI uptake with lung function analyses and HRCT findings.^[19]^ These results indicate that molecular fibroblast activation correlates with fibrotic tissue remodeling and disease progression in SSc-ILD, introducing the prospect of using FAPI for risk stratification of patients with fibrotic lung disease. Employment of 68Ga-FAPI-04-PET/CTs outside of SSc proved to be fruitful, as shown in the work of Röhrich *et al*., which demonstrates the potential of FAPI-PET to differentiate ILD from concomitant lung cancer by the different signal patterns.^[20]^ Studies focusing on other ILD entities have highlighted similar results: bronchioalveolar lavage fluids of IPF patients have higher FAP concentrations, which were then correlated with disease progression.^[18]^ A cut-off value of 192.5 ng/L for FAP bronchoalveolar lavage fluid (BALF) levels has been proposed, as these patients were at risk for a worse outcome.^[18]^

A study including a total of 83 ILD patients of different etiologies (IPF, idiopathic nonspecific interstitial pneumonia (NSIP), connective tissue disease (CTD), and interstitial pneumonia with autoimmune features (IPAF)) offers robust data of 68Ga-FAPI-04 performance in the clinical setting.^[21]^ The vast majority of patients included in this study ( > 90%) were treated with pirfenidone, an antifibrotic agent. Average radiotracer uptake did not differ between IPF and non-IPF patients, but median values of the total uptake were significantly higher in the former group, suggesting that the pathophysiological pathway of fibrosis progression in IPF might differ slightly from that of non-IPF. The median values of total uptake were also associated with serum Krebs von den Lungen-6 antigen (KL-6), a marker indicative of ILD severity.^[21]^ Lung fibrosis as a result of paraquat poisoning was also associated with FAPI uptake.^[22]^

68Ga-FAPI-04-PET/CT may enable quantification of fibrotic activity in other fibrotic organs as well. In a proof-of-concept study, our group could demonstrate that FAPI uptake in the myocardial tissue of SSc patients correlated with cardiac disease activity, while sequential 68Ga-FAPI-04-PET/CTs provided an edge over cardiac MRI (cMRI) in detecting ongoing injury.^[23]^ A distinctively higher uptake of FAPI was observed in SSc patients with arrhythmias, increased serum N-terminal Brain natriuretic Peptide (NT-pro-BNP) and higher late gadolinium enhancement on cMRI.^[23]^ These initial findings provide evidence of the potential use of 68Ga-FAPI-04-PET/CT in the evaluation of cardiac fibroblast activity and tissue response. Myocardial fibrosis as a result of myocardial infarction (MI) exhibited similarly significant FAPI uptake, which is in concordance with the current understanding of post-MI sequelae formation.^[24,25]^

## Current Limitations for Broadscale Implementation

The use of FAP tracer in ILD management is emerging as a novel approach for monitoring active fibrotic remodeling, providing valuable insight into the effectiveness of individual ILD treatment. However, broadscale implementation of 68Ga-labeled FAPI-PET is currently still hampered by the inherent limitations of tracer production: Gallium generators are generally needed for the on-site production of radioisotope due to the relatively short half-life of 68Ga (68 min). This enables an independent production of radioisotope, but results in limited yield for only a few possible examinations per day.^[26]^ Consequently, 68Ga-labeled FAPI-PET would be better suited for institutions with their own production unit independent of centralized supply. The use of the longer-lived ^18^F-FAPI compounds provides a convenient alternative. Mori *et al*. have demonstrated the efficacy of ^18^F-FAPI-74 PET/ CT in a small cohort of patients with IPF, suggesting the possibility of an improved image resolution achieved via ^18^F-FAPI-PET.^[27]^ However, a direct comparison between ^18^F-and 68Ga-labeled FAPI is currently still lacking, as the majority of FAPI-PET studies have used 68Ga-labeled compounds. In this context, it is also noteworthy to acknowledge that different FAP tracers, such as FAPI-02, FAPI-46 and FAPI-74, have different biodistributive and pharmacokinetic characteristics, and thus studies using different compounds should also be compared with caution.^[28]^

## Alternative Tracers for Pulmonary Fibrosis

Currently, FAP tracers stand in the focus of intensive research, as one of the most promising PET tracers in imaging fibrosis. Incidental findings in larger pulmonary fibrosis cohorts suggest that other tracers might be also interesting for future imaging options in SSc, such as 89Zr-rituximab targeting CD20-positive B lymphocytes and ^18^F-FB-A20FMDV2 targeting αvβ6, an integrin implicated in pulmonary fibrosis.^[29,30]^

Several PET markers tested in preclinical IPF models have shown promising results, suggesting potential application in SSc-ILD. In a murine model, 64Cu-radiolabeled platelet glycoprotein roman 6 (VI) fusion protein (64Cu-GPVI-Fc) was shown to detect active tissue fibrosis, but not the initial inflammatory reaction, providing a potential modality of discerning the two pathologic processes.^[31]^ A preclinical proof-of-concept study has demonstrated that expression of integrin αvβ3 and somatostatin receptor 2 (SSTR2) is upregulated in ILD, and that these proteins could serve as targets for specifically labeled tracers.^[32]^ Other tracers that have shown promising results in IPF imaging include cysteine cathepsins ^[33]^ and C-C chemokine receptor type 2 (CCR2),^[34]^ both of which are expressed on activated macrophages. A broad range of tracers directed against other pathophysiological components of ILD progression are also currently under investigation.^[35]^ These are, however, still in their incipient phase and require further validation.

## Future Perspectives

An increasing number of studies provide evidence that FAPI-PET/CT can quantify fibrotic activity in patients with fibrotic remodeling of the lung parenchyma. Its clinical applications are increasingly evident, ranging from assessing disease activity to monitoring therapy effectiveness and supporting patient stratification. As the field of molecular imaging is rapidly developing, this is the ideal moment to establish the tracer of choice and standardize methodological aspects before widespread adoption in the clinical practice. Finally, PET/CT imaging offers clinicians insight into an otherwise difficult to evaluate process, namely active fibrotic tissue remodeling, and may help to optimize the management of patients with ILD and other fibrotic conditions.
